# *In Vitro* Effect of 5-Nitroimidazole Drugs against Trichomonas vaginalis Clinical Isolates

**DOI:** 10.1128/spectrum.00912-22

**Published:** 2022-07-13

**Authors:** Andile Mtshali, Sinaye Ngcapu, Kavitha Govender, A. Willem Sturm, Prashini Moodley, Bronwyn C. Joubert

**Affiliations:** a School of Laboratory Medicine and Medical Science, Department of Medical Microbiology, University of KwaZulu-Natal, KwaZulu-Natal, South Africa; b Centre for the AIDS Programme of Research in South Africa, Durban, South Africa; Keck School of Medicine, University of Southern California

**Keywords:** *Trichomonas vaginalis*, susceptibility testing, nitroimidazoles, metronidazole, *in vitro*

## Abstract

Infections with the sexually transmitted parasite Trichomonas vaginalis are normally treated with metronidazole, but cure rates are suboptimal and recurrence rates following treatment are high. Therefore, our objective was to assess the *in vitro* antitrichomonas activities of three other 5-nitroimidazole drugs and compare them with metronidazole. T. vaginalis isolates (*n* = 94) isolated from South African women presenting with vaginal discharge syndrome at two sexually transmitted disease clinics in KwaZulu-Natal were grown from frozen stock. Twofold serial dilutions (16 to 0.25 mg/L) of metronidazole, tinidazole, ornidazole, and secnidazole were prepared in Diamond’s broth. The MICs were read after 48 h of anaerobic incubation at 37°C. An MIC of <2 mg/L was defined as susceptible, an MIC of 2 mg/L was defined as intermediate, and an MIC of >2 mg/L was defined as resistant. Sixty-one percent (57/94) of the T. vaginalis isolates were susceptible to metronidazole, 80% (75/94) were susceptible to tinidazole, 75% (71/94) were susceptible to secnidazole, and 89% (84/94) were susceptible to ornidazole. Resistance levels were 11%, 2%, and 1% for metronidazole, tinidazole, and secnidazole, respectively, while no resistance was observed for ornidazole. Intermediate scores were 28% for metronidazole, 18% for tinidazole, 24% for secnidazole, and 11% for ornidazole. Isolates from a proportion of women with bacterial vaginosis (BV) had higher MICs, and no isolates from women coinfected with another sexually transmitted infectious organism were resistant to any of the antimicrobials tested. This study showed that among T. vaginalis isolates in KwaZulu-Natal, there is no *in vitro* resistance to ornidazole. Of the 5-nitroimidazoles, metronidazole showed the highest level of resistance. The very low levels of resistance for the other three antimicrobials indicate that all three are viable options as a replacement for metronidazole if these *in vitro* findings are found to correlate with clinical outcomes.

**IMPORTANCE** Trichomonas vaginalis is the most common nonviral sexually transmitted infection associated with reproductive sequelae and HIV acquisition risk worldwide. Despite its role in reproductive health, a high prevalence in South Africa, and the reported metronidazole resistance worldwide, no alternative regimens have been tested against T. vaginalis in our setting. This study compared the susceptibility patterns of three other 5-nitroiminazoles (secnidazole, tinidazole, and ornidazole), which are active against T. vaginalis with metronidazole *in vitro*. Metronidazole, the drug of choice for the treatment of trichomoniasis, showed the highest level of resistance, while the three regimens showed very low levels of resistance. These data indicate that all three are viable options as a replacement for metronidazole if these *in vitro* findings are found to correlate with clinical outcomes.

## INTRODUCTION

Trichomonas vaginalis is the causative agent of urogenital trichomoniasis in women and men (1, 2). In 2020, the WHO estimated the number of new trichomoniasis cases globally was 156.3 million, with Africa accounting for 12% of the global prevalence (1). South Africa has a high burden of T. vaginalis infection among women, with a reported prevalence of 4.6 to 20% (2, 3). T. vaginalis infection is often asymptomatic; however, when symptoms arise, women present with vaginal odor, yellowish-green discharge, vulval itching, and less frequently, a strawberry cervix (4).

**FIG 1:**
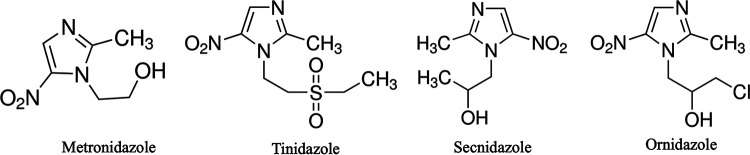
Structural differences between the side chains of four 5-nitroimidazoles compounds active against T. vaginalis. Adopted from Miyamoto et al. (10).

Trichomoniasis is associated with complications such as pelvic inflammatory disease, cervical erosion, cervical cancer, infertility, and increased risk of acquiring HIV (5). Infections with T. vaginalis are treated with a single dose of metronidazole, a 5-nitroimidazole compound. In case of failure, a multiple-dose regimen over 5 days is applied (6). The single dose of metronidazole is usually well tolerated, but mild gastrointestinal side effects do occur. These side effects are more frequent with multiple doses (7). Of the seven compounds in the 5-nitroimidazole class, tinidazole, secnidazole, and ornidazole have shown activity against T. vaginalis (8). These drugs have an identical mechanism of action, but their pharmacokinetic properties are different due to the chemical substitutions on the side chain (Fig. 1) (9, 10). Only metronidazole and tinidazole are approved by the U.S. Food and Drug Administration for the treatment of trichomoniasis (11). Tinidazole, although superior to metronidazole, is not available in the public health care setting in South Africa due to the high costs.

While metronidazole is usually effective in treating T. vaginalis infections, *in vitro* resistance and clinical failure have been widely reported (12, 13). This failure to cure trichomoniasis is of concern, especially in limited-resourced countries where no alternative is available. Persistent infections can be the result of treatment failure but also of reinfection by the usually untreated partner (14, 15). Since sexually transmitted infections are managed syndromically in South Africa (16) and other low-income countries, microbiological surveillance with antimicrobial susceptibility testing is required periodically to identify changes in the prevalence of circulating organisms and their susceptibility profiles. The purpose of this study was to compare the inhibitory properties of metronidazole, tinidazole, secnidazole, and ornidazole against T. vaginalis isolates from women presenting with vaginal discharge to sexually transmitted disease clinics in KwaZulu-Natal, South Africa.

## RESULTS

**FIG 2:**
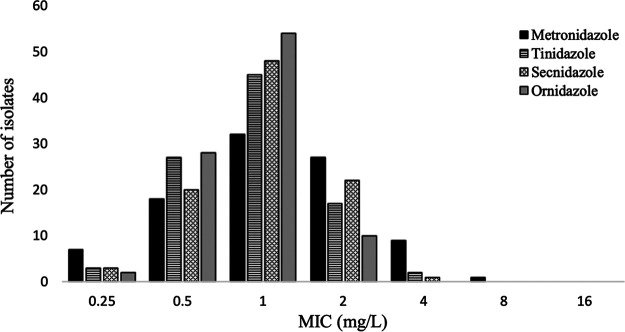
Distribution of MIC values of metronidazole, tinidazole, secnidazole, and ornidazole for T. vaginalis isolates (*n* = 94). The *y* axis represents the number of T. vaginalis isolates, and the *x* axis represents the MICs of the corresponding drugs.

For all T. vaginalis isolates, *in vitro* susceptibility tests were performed to determine the MICs of four 5-nitroimidazole drugs (17). After 48 h of incubation, the mean (± standard deviation [SD]) MIC of metronidazole was 2.25 (±2.8) mg/L, for both tinidazole and secnidazole the MIC was 1.11 mg/L (±1.5), and for ornidazole the MIC was 0.5 (±0.7) mg/L. Figure 2 shows the MIC distributions for the four compounds. Isolates classified as resistant (MICs of >2 mg/L) were 11% for metronidazole, 2% for tinidazole, 1% for secnidazole, and 0% for ornidazole. Intermediate scores for the four drugs (i.e., an MIC of 2 mg/L) were found in 29%, 18%, 23%, and 0% of isolates, respectively. Susceptibility was identified in 61% of isolates for metronidazole, 80% for tinidazole, 75% for secnidazole, and 89% for ornidazole. As expected, the MIC for Bacteroides fragilis of metronidazole and tinidazole was 4 mg/L, while Propionibacterium acnes was resistant at the highest drug concentrations tested in this study, with an MIC of >256 mg/L. We used these bacteria to confirm that there were no calculation or dilution errors.

The cumulative percentages of susceptibility at the different concentrations for the four compounds are given in Table 1. The MIC_90_ was 2 mg/L for metronidazole, 1 mg/L for ornidazole, and between 1 and 2 mg/L for tinidazole and secnidazole.

**TABLE 1:** Cumulative percentages of MICs of the four 5-nitrimidazoles against 94 T. vaginalis isolates

5-Nitroimidazole	Cumulative % of MIC (mg/L)						
	0.25	0.5	1	2	4	8	16
Metronidazole	7	27	61	89	99	100	
Tinidazole	3	32	80	98	100		
Secnidazole	3	24	76	99	100		
Ornidazole	2	32	89	100			

**FIG 3:**
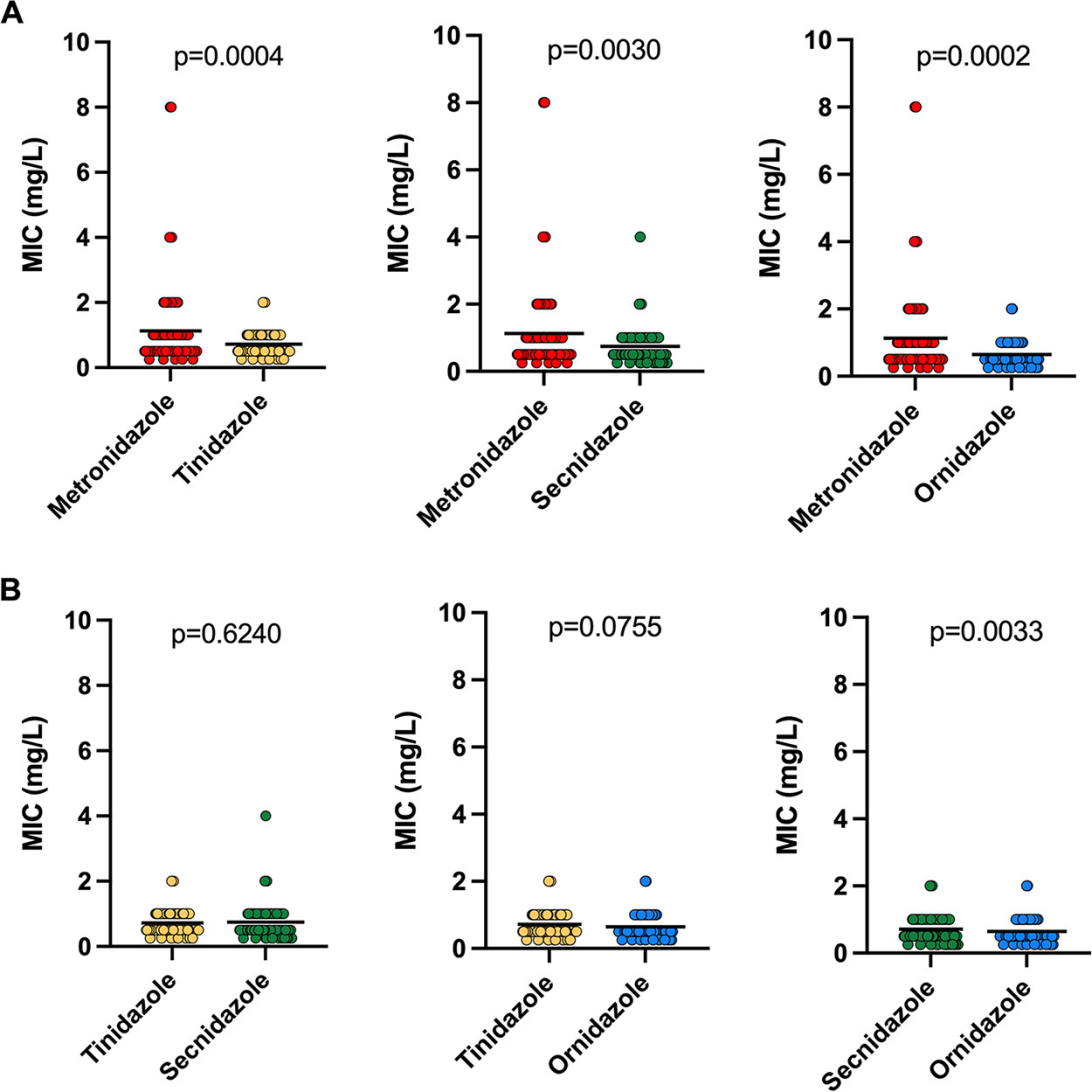
MICs for T. vaginalis isolates (*n *= 94) against four 5-nitroimidazoles. MICs of tinidazole, secnidazole, and ornidazole were significantly lower than that of metronidazole (A), and secnidazole MICs were significantly higher than those of ornidazole (B). A paired *t* test was used to compute *P* values (indicated above the graph). The solid line indicates the mean.

The MICs of metronidazole were compared with those for the other three compounds (Fig. 3A). MICs of tinidazole, secnidazole, and ornidazole were significantly lower than those of metronidazole (metronidazole versus tinidazole, *P* = 0.0004; metronidazole versus secnidazole, *P* = 0.0030; metronidazole versus ornidazole, *P* = 0.0002). When we compared the other three compounds with each other (Fig. 3B), a difference in MICs was only observed between secnidazole and ornidazole (*P* = 0.0033). We next determined if the MIC distribution differed in women co-infected or not with another sexually transmitted organism or bacterial vaginosis (BV) was associated with any high MIC in all compound. There was no significant association between women coinfected with any of these antimicrobials (Table 2). However, of the 11 women infected with T. vaginalis isolates with a metronidazole MIC of >2 mg/L, 5/11 (45%) had a Nugent score compatible with BV, while 6/11 (54%) had intermediate BV.

**TABLE 2:** MIC distributions and comparisons of T. vaginalis isolates from women coinfected or not with another sexually transmitted organism and/or BV

Antimicrobial	Coinfection	Extent of coinfection	No. of isolates with MIC (mg/L) of:							*P* value^*a*^
			0.25	0.5	1	2	4	8	16	
Metronidazole	Bacterial vaginosis	No BV	1	3	4	0	0	0	0	0.3665
		Intermediate BV	1	19	8	5	5	0	0	
		BV	3	18	19	8	4	1	0	
	*Neisseria gonorrhoea*	Yes	1	6	10	2	0	0	0	0.7726
		No	4	34	21	11	3	0	0	
	Chlamydia trachomatis	Yes	0	7	5	1	0	0	0	0.5683
		No	5	33	25	11	3	0	0	
Tinidazole	Bacterial vaginosis	No BV	1	4	3	0	0	0	0	0.3749
		Intermediate BV	3	19	11	1	1	0	0	
		BV	4	24	22	2	1	0	0	
	*Neisseria gonorrhoea*	Yes	1	11	7	0	0	0	0	0.7511
		No	7	36	29	3	0	0	0	
	Chlamydia trachomatis	Yes	1	8	4	0	0	0	0	0.4641
		No	7	38	31	3	0	0	0	
Secnidazole	Bacterial vaginosis	No BV	3	2	3	0	0	0	0	0.4380
		Intermediate BV	4	12	14	3	1	0	0	
		BV	7	25	20	0	0	0	0	
	*Neisseria gonorrhoea*	Yes	2	6	10	1	0	0	0	0.2008
		No	12	33	27	2	1	0	0	
	Chlamydia trachomatis	Yes	2	5	6	0	0	0	0	>0.9999
		No	12	17	29	3	1	0	0	
Ornidazole	Bacterial vaginosis	No BV	1	5	2	0	0	0	0	0.7250
		Intermediate BV	4	18	10	2	0	0	0	
		BV	6	31	15	0	0	0	0	
	*Neisseria gonorrhoea*	Yes	3	9	7	0	0	0	0	0.8827
		No	8	45	20	2	0	0	0	
	Chlamydia trachomatis	Yes	2	8	3	0	0	0	0	0.4836
		No	9	22	24	2	0	0	0	

a The Mann-Whitney U test was used to compare negative and positive groups; *P* values of <0.05 were considered statistically significant.

## DISCUSSION

For decades, metronidazole was considered the drug of choice for the treatment of trichomoniasis. Patients that do not respond to the standard dose of 500 mg in a single dose have been reported in most settings. Such refractory cases that fail multiple attempts with the standard dose are treated with a double dose, with prolonged courses of multiple daily doses of metronidazole or with tinidazole (13, 17, 18). However, higher doses of metronidazole often result in side effects, such as vomiting and dizziness, as does prolonged treatment, and tinidazole is not readily available in most countries (13). With several 5-nitroimidazoles being reported to have antitrichomonal activity (18, 19), knowledge of the MIC distribution for isolates obtained in local settings to these drugs may provide useful information in the choice of alternative treatment in refractory cases. In South Africa, tinidazole is unavailable since it has not been registered. The data presented here could help in motivating registration of this or any of the other compounds for the treatment of trichomoniasis that does not respond to metronidazole.

The data from this study support the findings that demonstrated that all three alternative 5-nitroimidazoles had better activity against T. vaginalis *in vitro* than did metronidazole. Ornidazole was the only compound against which no resistance was observed. This supports the finding of others that ornidazole is the superior drug for the treatment of trichomoniasis (19, 20). Single-dose treatment with ornidazole resulted in 100% trophozoite eradication after 1 week, compared to 95% eradication for the same dose of tinidazole (19).

If the rule for bacterial infections, that an antimicrobial agent should not be used as first-line treatment if the MIC_90_ is above the breakpoint for resistance, is also applicable for T. vaginalis, then in KwaZulu-Natal, metronidazole with an MIC_90_ of 2 mg/L should no longer be used for that purpose. However, whether this rule is applicable and whether 2 mg/L is the true breakpoint is unknown.

Also, how metronidazole MICs correlate to clinical response is not clear. Treatment failure of infections with susceptible isolates is not uncommon. Several explanations for this have been postulated. Since infection in men is usually asymptomatic, partners often remain untreated. Early reinfection by a stable or a casual partner of the female then presents as treatment failure. Other possibilities are pharmacokinetic differences between women and reduction in the availability of the drug in the vagina by anaerobic bacteria, which are also susceptible to metronidazole. Several studies have attributed the reduced efficacy of metronidazole to the presence of diverse microbial communities that metabolize the drug (17, 21, 22). Combined clinical, epidemiological, and *in vitro* studies that address the above issues in the same group of patients are needed to provide the answers.

All isolates tested were from symptomatic women. A proportion of the women were also infected with a bacterial pathogen; however, this was not associated with increased susceptibility to any compound tested. How much each of these pathogens contributed to the symptoms is unknown. Although it is unlikely that T. vaginalis in asymptomatic women and men differs from that in symptomatic individuals in the same population, studies that include asymptomatics should be considered.

As with all *in vitro* susceptibility tests, the T. vaginalis isolates were passaged several times before the tests. This could have altered the phenotype. Bacterial controls were used instead of T. vaginalis isolates with a known susceptibility profile. Controls with a known MIC are used to test the adequacy of the dilution series. Since the bacteria were tested under the same incubation circumstances and in the same medium, this should be acceptable. Finally, we did not determine MICs under aerobic conditions, and the aerobic assessment may have complemented the anaerobic assay.

While metronidazole has been the standard of treatment for trichomoniasis for many years (12, 13, 17), our study demonstrated the highest increase in an MIC for T. vaginalis isolates was to metronidazole, followed by tinidazole and secnidazole, and there were no increased MICs to ornidazole. This suggests that ornidazole might be the best alternative if clinical resistance to metronidazole treatment is observed. With all infections, the predictive value of susceptibility tests is not 100%, including trichomoniasis. Some patients with trichomoniasis infected with an *in vitro* “resistant” organism respond well to standard metronidazole therapy, while others with a “susceptible” organism fail to respond (23–26). Further studies are needed to better understand clinical treatment outcome in the context of *in vitro* susceptibility profiles of the 5-nitroimidazoles.

## MATERIALS AND METHODS

### Trichomonas vaginalis isolates and controls.

The study included 94 T. vaginalis isolates obtained during a former study on the etiology of vaginal discharge syndrome in KwaZulu-Natal (27). Infections with Chlamydia trachomatis and Neisseria gonorrhoeae (gonococcus) were diagnosed using a BD ProbeTec ET assay (Becton, Dickinson, Sparks, MD). Bacterial vaginosis was diagnosed microscopically using Nugent’s score (28). The median age of the women from whose vaginal specimens these isolates were grown was 27 years with an interquartile range (IQR) of 23 to 34 years. Thirteen of the 94 were coinfected with Chlamydia trachomatis and 19 were coinfected with Neisseria gonorrhoeae. Based on the Nugent Gram stain score, 55 had BV (scores of 7 to 10) (28). T. vaginalis isolates were cryopreserved in Diamond’s medium supplemented with 15% heat-inactivated fetal bovine serum and 10% dimethyl sulfoxide and slowly reducing the temperature to −85°C.

Bacteroides fragilis (ATCC 25285) and Propionibacterium acnes (ATCC 11827) were used as susceptible and resistant controls, respectively. The MIC in Diamond’s medium of the B. fragilis strain to metronidazole and tinidazole was 4 mg/L, which is within the published value range in bacterial growth medium (29). The MICs for metronidazole and tinidazole of the P. acnes strain were >256 mg/L (30). Since no published MIC values for secnidazole and ornidazole are available, these MICs were determined experimentally three times in triplicate. The mean values obtained were used as a reference for the drug in the tests. The study was approved by the Biomedical Research Ethics Committee of the University of KwaZulu-Natal (BE220/13).

### Drug susceptibility testing.

Cryopreserved cultures were placed in a water bath of 37°C until thawed. Immediately upon thawing, the isolates were transferred to 5 mL drug-free Diamond’s medium and incubated at 37°C. Viability was confirmed microscopically after 24 h before subculturing. When motile trichomonas cells were observed in these subcultures, broths were centrifuged at 500 × *g* for 5 min, and the pellet was resuspended in Diamond’s medium for use in drug susceptibility tests.

The MICs of metronidazole, tinidazole, secnidazole, and ornidazole (Sigma-Aldrich, USA) were determined for each isolate using a broth microdilution assay under anaerobic conditions. Twofold serial dilutions (32 to 0.25 mg/L) of each of the 5-nitroimidazole compounds were prepared in flat-bottomed 96-well microtiter plates using Diamond’s medium as the diluent. Trichomonads were enumerated using a hemocytometer. Each well was inoculated with 3 × 10^3^ organisms. Plates were incubated for 48 h at 37°C in an airtight sealed container with an AnaeroGen sachet (Oxoid, Hampshire, United Kingdom) and an anaerobic indicator strip (Oxoid). The results were evaluated after 48 h of incubation at 100× magnification using an inverted phase-contrast microscope (Olympus IXZ-SLP). Turbid media indicated “growth,” whereas clear media indicated “no growth” of the control organisms (Bacteroides fragilis ATCC 25285 and Propionibacterium acnes).

The growth in each nitroimidazole dilution was assigned a score as described by the Upcroft group (Table 3) (17). The MIC was defined as the lowest drug concentration that produced a score of 1+. Before incubation, the score in each well was 1+; therefore, a score of 1+ after incubation indicated inhibition of replication. Experiments were conducted in duplicate. If different MICs were observed or if the test isolate had an MIC of >2 mg/L, the experiment was repeated, and the mean of the four values was used. Based on the results, isolates were classified as follows: MIC < 2 mg/L, susceptible; MIC = 2 mg/L, intermediate; MIC > 2 mg/L, resistant (17). The controls for the assay included a growth control in drug-free medium as well as MIC determinations for both control organisms in Diamond’s medium.

**TABLE 3:** Assessment of MICs for T. vaginalis isolates^*a*^

Observation category	Score
0–10 motile parasites; ≤20% coverage of well surface	1+
>10 motile parasites; 20–50% coverage of well surface	2+
>50 % coverage of well surface (i.e., almost confluent growth with much motility)	3+
Confluent growth with full motility	4+

a Observations of growth in each nitroimidazole dilution were assigned a score as described by the Upcroft group (17).

### Statistical analyses.

The Statistical Package for the Social Sciences (SPSS Inc., v13.0) was used for analysis. Microsoft Excel was used to compute the plots. The categorical variables were summarized in the form of counts and percentage frequencies. A paired *t* test was used to compute the association between the compounds, and the Mann-Whitney U test was used to determine the association between the compounds for isolates from women coinfected with other sexually transmitted infectious organisms and/or BV and those with no coinfection. A *P* value of <0.05 was considered statistically significant.
